# Zooming in on what counts as core and auxiliary: A case study on recognition models of visual working memory

**DOI:** 10.3758/s13423-024-02562-9

**Published:** 2024-09-17

**Authors:** Maria M. Robinson, Jamal R. Williams, John T. Wixted, Timothy F. Brady

**Affiliations:** 1 Department of Psychology, University of Warwick, Coventry, UK; 2 Department of Psychology, Yale University, New Haven, CT USA; 3 Department of Psychology, University of California, San Diego, CA USA

**Keywords:** Metascience, Measurement, Auxiliary assumptions, Memory, Visual working memory limits, K capacity, Discrete-slot models, Signal detection models

## Abstract

Research on best practices in theory assessment highlights that testing theories is challenging because they inherit a new set of assumptions as soon as they are linked to a specific methodology. In this article, we integrate and build on this work by demonstrating the breadth of these challenges. We show that tracking auxiliary assumptions is difficult because they are made at different stages of theory testing and at multiple levels of a theory. We focus on these issues in a reanalysis of a seminal study and its replications, both of which use a simple working-memory paradigm and a mainstream computational modeling approach. These studies provide the main evidence for “all-or-none” recognition models of visual working memory and are still used as the basis for how to measure performance in popular visual working-memory tasks. In our reanalysis, we find that core practical auxiliary assumptions were unchecked and violated; the original model comparison metrics and data were not diagnostic in several experiments. Furthermore, we find that models were not matched on “theory general” auxiliary assumptions, meaning that the set of tested models was restricted, and not matched in theoretical scope. After testing these auxiliary assumptions and identifying diagnostic testing conditions, we find evidence for the opposite conclusion. That is, continuous resource models outperform all-or-none models. Together, our work demonstrates why tracking and testing auxiliary assumptions remains a fundamental challenge, even in prominent studies led by careful, computationally minded researchers. Our work also serves as a conceptual guide on how to identify and test the gamut of auxiliary assumptions in theory assessment, and we discuss these ideas in the context of contemporary approaches to scientific discovery.

## Introduction

What does it take to falsify a psychological theory? Researchers may have a stock response to this question: Identify a core prediction of the target theory, formulate a competing hypothesis, and design an experiment to test between them. If the evidence favors the competing hypothesis, the theory is falsified. Unfortunately, most researchers also know that this routine practice can be notoriously difficult to implement. As pointed out by Meehl (2004) over four decades ago (1978), it can be hard to ‘kill’ a psychological theory. This point has been made again recently in different guises following the reported replication crisis in the behavioral sciences (e.g., Ioannidis, 2005; Pashler & Harris, 2012; Simmons et al., 2011; Open Science Collaboration, 2015), which sparked renewed interest in increasing the rigor of theory development and testing in psychology (e.g., Davis-Stober, & Regenwetter, 2019; Kellen et al., 2021a, 2021b; Grahek et al., 2021; Guest & Martin, 2021; Navarro, 2021; Oberauer & Lewandowsky 2019; Regenwetter & Robinson, 2017; Regenwetter et al., 2022a, 2022b; Wilson et al., 2022). So, how can the routine practice of scientific inquiry fail? What are some major hurdles for theory testing in psychology, and what steps can be taken to overcome them?

Testing theories is deceptively difficult because they inherit a new set of assumptions as soon as they are linked to a specific experiment (Kellen et al., 2021a, 2021b; Tal, 2013; Trafimow, 2012). These assumptions are typically made at the discretion of the researcher because he or she needs to determine which predictions are core to a theory and which are not, validate methodology, and ensure that analytic methods are robust against imprecise measurements and noisy data (Lakatos, 1976; Strevens, 2020). Most of these assumptions are ancillary to the theory – meaning they do not follow from it directly – but they are requisite for testing the theory in practice. Furthermore, because theories can be linked to an experiment in a variety of ways (Scheel et al., 2021; Tal, 2013), researchers are left with the challenging conceptual task of identifying and evaluating auxiliary assumptions that are made at different (technical and conceptual) stages of theory testing and different (theory-specific and theory-general) levels of the theory itself. Recent articles by Starns et al. (2019) and Dutilh et al. (2019) point to the severity of these challenges by demonstrating that cognitive modelers can reach completely opposite conclusions even when given an opportunity to analyze exactly the same data.

The goal of our exposition is to extend on this line of work by focusing on an accessible case study of how to identify and evaluate auxiliary assumptions. We also build on existing metatheoretical literature by providing a concrete example on the potentially long-term consequences of failing to assess auxiliary assumptions on theorizing and measurement within a prominent research domain in cognitive psychology. We do this through a conceptual and technical reanalysis of an existing, high-profile study on recognition models of visual working memory (Rouder et al., 2008) as well as its replications (Donkin et al., 2014).

Rouder et al. (2008) reported evidence for a classic “all-or-none” model of visual working memory, according to which memoranda are stored with complete fidelity or not at all. More than a decade after the paper’s publication, these results continue to be referenced as support for all-or-none models as well as the view that visual working memory capacity is limited to approximately “three to four” fixed representations (e.g., Cowan, 2001; Forsberg et al., 2021; Jakubowska et al., 2021; Kardan, et al., 2020; Kvitelashvili & Kessler, 2024; Medernach et al., 2023; Pratte & Green, 2023; Roark et al., 2023; Strzelczyk et al. 2023), which still pervades nearly all popular understanding of individual differences in visual working memory (e.g., Cowan, 2014; Green & Pratte, 2022; Luck & Vogel, 2013; Ngiam et al., 2023). While alternative approaches to visual working memory measurement exist (e.g., continuous reproduction tasks), many researchers continue to use change detection tasks, and, even in the most high-profile situations (e.g., a large-scale collaboration: Strzelczyk et al., 2023), these researchers continue to use measures based primarily on discrete-slot models like those supported by Rouder and colleagues.

Importantly, as we review below, the results of Rouder et al. (2008) and the study’s replications directly conflict with evidence against all-or-none models of visual working memory found using other methods, like continuous reproduction tasks. This raises the key question: *Does this study reveal a theoretically meaningful difference in how people store memory representations across task demands (change detection vs.* *continuous reproduction), or are the results an artifact of untested auxiliary assumptions*? We find support for the latter view. That is, after evaluating key auxiliary assumptions, we find evidence for the completely opposite conclusion, that is, that continuous resource models outperform all-or-none models of visual working memory. Together, our reanalysis illustrates that even for mathematically well-specified models that make qualitatively distinct predictions, tested by extremely quantitatively savvy researchers, it can be a significant challenge to disentangle what counts as core and auxiliary. Using these articles as a case study, we offer concrete examples on how to identify and test auxiliary assumptions at different stages of study design and analysis, as well as at different levels of a psychological theory.

We highlight that we focus on the Rouder et al. (2008) article and its replications because of its high impact, underscoring the seriousness of these issues, and because it fits with our expertise on models of memory, giving us an appropriate vantage point for critically evaluating it. Furthermore, the question of whether working-memory representations are fundamentally all-or-none or continuous has been a major aspect of working-memory research for over two decades (e.g., Bays & Husain, 2008; Luck & Vogel, 1997; Zhang & Luck, 2008), and speaks to many larger issues about the nature of cognitive architecture. For instance, it relates to questions of whether representations are more discrete versus distributed, which have been a core aspect of cognitive science since the 1950s (e.g., Garnelo & Shanahan, 2019; Marcus, 1998; Rosenblatt, 1958; Rumelhart et al., 1988). However, the issues we overview are in no way unique to these articles or this research domain, and we provide other examples across psychology in the *General discussion*. As part of this review, we also integrate and build on recent conceptual and technical discussions of best practices in theory assessment (e.g., Kellen et al., 2021a; Scheel et al., 2021; Zilker, 2022). Together, our article is intended for a broad audience, with a range of expertise and interests in meta-theoretic issues in psychology. In the following section, we summarize the relevant background of our case study article by Rouder et al. (2008) and Donkin et al.’s (2014) replication of this work.

### Recognition theories of visual working memory

Visual working memory is a fundamental memory system that supports our ability to recognize objects (e.g., Emrich et al., 2011), maintain a stable sense of the environment across eye movements (e.g., Irwin, 1991), and keep active mental representations in the service of goals (e.g., McCants et al., 2020). In addition to playing a key role in everyday function, visual working memory limits are associated with other global markers of cognitive function, such as general intelligence (Luck & Vogel, 2013). For these reasons, a large body of research focuses on developing theories and measures of visual working memory processes and architecture, and testing these via computational models (e.g., Bays et al., 2011; Oberauer & Lin, 2017; Rouder et al., 2008; Schurgin et al., 2020; Van den Berg et al., 2014; Zhang & Luck, 2008).

**Fig. 1:**
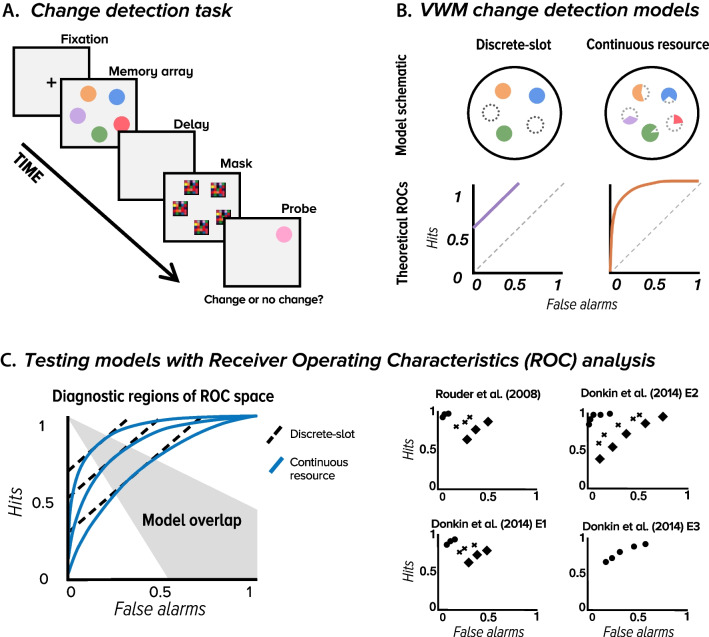
(**A**) An example trial in a change detection task in which participants need to remember five colored squares and their spatial locations. After a brief delay, participants must indicate whether the probed item is the same or different than the item originally presented at that location. (**B**) A schematic of all-or-none and continuous resource models, as well as their theoretical receiver operating characteristics (ROCs). Classic discrete slot models postulate that memory fails in an all-or-none way and predicts a linear ROC. Continuous resource models postulate that memory representations are continuous and predict curvilinear ROCs. (**C**). These models make qualitatively distinct predictions about the shape of the ROC, but there is a portion of ROC space where the models make overlapping predictions (gray-shaded region), making data that falls in this region non-diagnostic. As shown in the aggregate ROC data, experiments that use only a few (e.g., three) base-rate manipulations may generate data that falls in this non-diagnostic region

One of the most prominent visual working memory tasks is the recognition memory, *change detection* task, in which participants respond on the presence or absence of a change to a probed item (Fig. 1A). This task was one of the first used to measure limits in visual working memory capacity (Luck & Vogel, 1997; Pashler, 1988), and continues to be popular because it provides an easy way of probing visual memory as a function of experimental conditions or individual differences both in normal (e.g., Awh et al., 2007; Fukuda et al., 2010; Xu & Chun, 2006) and in clinical populations (e.g., Oudman et al., 2020). In light of its prevalence, it is important to find a theory that best characterizes change detection performance to guide theorizing and measurement in the visual working memory domain.

Two major theories of recognition visual working memory have been relied on for measurement of performance in change detection tasks, the all-or-none and continuous resource theory (Fig. 1B). All-or-none theories postulate that visual working-memory limits are set in terms of a discrete number of “slots” that store representations of simple or bound features (e.g., Cowan, 2001; Pashler, 1988; Vogel et al., 2001), whereas continuous resource theories postulate that visual working memory limits are set in terms of a continuous resource that is distributed across features and items (e.g., Alvarez & Cavanagh, 2004; Schurgin et al., 2020; Van den Berg et al., 2014; Wilken & Ma, 2004). A formal way of distinguishing these two specific models – which have dominated in change detection tasks – is via Receiver Operating Characteristic (ROC) analysis, a central modeling approach in the study of recognition memory (e.g., Wixted, 2007; Yonelinas & Parks, 2007) in which hits are plotted as a function of false alarm rates for different levels of a person’s response bias.

All-or-none and continuous resource models make qualitatively different predictions of how hit (“present” responses on present trials) and false alarm (“present” responses on absent trials) rates vary with changes in response bias (propensity to say “present”) in change detection tasks (Fig. 1C). All-or-none models postulate that there is a fixed probability that an item is or is not in memory, and response bias only affects the probability of guessing that an item is present. This entails a linear change in hits as a function of false alarms since bias is considered to affect hits and false alarms via a change in a fixed slope. In contrast, traditional resource models postulate a Gaussian probability distribution over possible memory strengths. This entails that a shift in response bias predicts a curvilinear change in hits as a function of false alarms. Thus, if data fall within a diagnostic region of ROC space (Fig. 1C), these two models can be compared by assessing the relative fit of each function to the empirical ROC. This was the approach taken by Rouder and colleagues (2008), who applied ROC analysis and manipulated response bias via a base-rate manipulation by varying the proportion of change trials across experimental blocks.

A critical broad takeaway from our summary is that, in principle, testing between continuous resource and all-or-none models should be extremely straightforward because both are relatively simple computational models that make qualitatively distinct predictions about the shape of ROC functions. However, as we show, even such seemingly straightforward model comparisons, implemented by computational modeling experts, can be extremely difficult to implement rigorously in practice (also see, e.g., Dube & Rotello, 2012).

### Rouder et al.’s (2008) findings and intended scope of current reanalysis

Rouder et al. (2008) reported evidence for all-or-none models. These results continue to have a major impact on theorizing and measurement in the field of visual working memory. For instance, they are commonly cited as evidence for item-based limits within the visual working memory literature (Cowan, 2014; Luck & Vogel, 2013; Ngiam et al., 2023; Sone et al., 2021). Furthermore, they are used to motivate a measure, the “K” metric, that postulates all-or-none processing in change detection paradigms (Cowan, 2001; Rouder et al., 2011). Importantly, this metric is still commonly used in the study of how individual differences in visual working memory limits predict other higher-level processes, such as general intelligence (Fukuda, et al., 2010), reading ability (Daneman & Carpenter, 1980), and age-related cognitive changes (Jost et al., 2011). The continued use of K has major implications because it can capture some changes in performance as latent changes in visual working memory capacity, whereas resource model-based metrics capture these as changes in response bias (Williams et al., 2022). This implies that K may not simply be an imprecise but a biased measure of working memory capacity.

Importantly, the implications of our reanalysis and findings may not necessarily extend to other tasks; for instance, we are agnostic regarding whether our results extend to running memory span tasks, which use other stimuli (e.g., verbal stimuli) and alternative presentation formats (e.g., serial presentation). Furthermore, our results may not extend to other variants of item-limit models. In particular, many hybrid models and extensions to other tasks have been developed, most notably mixture models that incorporate elements of both slots and resources, which were first introduced in the visual working memory literature to account for data from continuous reproduction tasks (Zhang & Luck, 2008). While researchers have used ROC analyses to formally evaluate mixture models in change detection tasks (Robinson et al., 2020; Yonelinas, 2023; Xie & Zhang, 2017), there are currently no mixture-model based metrics of visual working memory limits that can be obtained without model fitting. Moreover, mixture models are conceptually and formally distinct from the class of all-or-none (“threshold”) models, which are supported by the results of Rouder et al. (2008). Given that mixture models share assumptions with both all-or-none and resource theory by postulating item limits and noisy memory representations, using K metrics as a proxy for mixture models does not have a principled basis. Nevertheless, the dominant measure of visual working memory item-limits in change detection tasks is still overwhelmingly analyzed using the classic all-or-none model (e.g., “K” values; Forsberg et al., 2021; Jakubowska et al., 2021; Kardan et al., 2020; Kvitelashvili & Kessler, 2024; Pratte & Green, 2023; Roark et al., 2023; Strzelczyk et al. 2023). The question of whether memories fail in an all-or-none manner in visual working memory, therefore, remains critical to theorizing and measurement in the recognition working memory domain.

To summarize, even though other models now exist for continuous reproduction tasks (e.g., Zhang & Luck, 2008), choosing between all-or-none and continuous resource metrics – which remain the two major ways of conceiving of performance in change detection tasks – has major consequences for theorizing and practice because they can yield qualitatively different and contradictory conclusions in real scenarios (e.g., Brady et al., 2022; Robinson et al., 2020; Williams et al., 2022). Furthermore, as we review next, although metrics based on discrete, all-or-none slots remain widely used in change detection (e.g., Strzelczyk et al., 2023), they have not received consistent support in other studies and paradigms.

### An exception in the visual working memory modeling literature

Despite their long-lasting impact, the findings of Rouder et al. (2008) are puzzling when viewed through the lens of contemporary modeling work and theorizing (Bays et al., 2009). This is because they conflict with modeling results from numerous other visual working memory experiments. First, these results conflict with evidence from a recent, novel critical test developed for change detection paradigms (Winiger et al., 2022), which was designed to provide an alternative, formal way of comparing all-or-none and resource models in change detection tasks, while obviating the limitations of relying on auxiliary assumptions of ROC-based modeling. This study used a response bias manipulation to examine whether low confidence judgments could be systematically biased, a result only consistent with resource models where confidence judgements are based on the relative evidence for one of two responses. This provides convergent evidence for graded rather than all-or-none information storage in change detection paradigms.

Second, the Rouder et al. (2008) results conflict with evidence from change detection paradigms in which ROCs are constructed via measures of confidence rather than manipulated via different base rates (Robinson et al., 2020; Williams et al., 2022; Wilken & Ma, 2004). Although the use of confidence-based ROCs has been criticized because, in principle, people can use complex, non-linear strategies to map memory states to confidence scales (Malmberg, 2002), this critique has not received consistent empirical support (Delay & Wixted, 2021). Moreover, as we previewed, ROCs built off base-rate manipulations also involve strong auxiliary assumptions, which can yield non-diagnostic data when violated (Macmillan & Creelman, 2005). At a minimum, inconsistent results across studies that involve basic modifications to task demands, such as use of confidence versus base-rate manipulations, signals a need to reassess methodological auxiliary assumptions.

Finally, the results of Rouder et al. contradict evidence from popular continuous reproduction tasks, in which participants use a continuous report, such as a color wheel, to make memory judgments (Wilken & Ma, 2004). Such continuous reproduction tasks yield distributions of memory errors, rather than discrete responses, making them more sensitive to potential variations in memory fidelity. Importantly, evidence from continuous reproduction tasks reveals consistent support for the view that visual memory representations are not all-or-none, but vary in precision as a function of memory load, encoding time and delay (e.g., Bays, 2015; Schurgin et al., 2020; Van den Berg et al., 2014; also see Zhang & Luck, 2008, who find evidence for variation in precision, rather than all-or-none memories like those compatible with all-or-none models of change detection tasks).

Given evidence against pure all-or-none models of visual working memory, what accounts for the continued impact of Rouder et al. (2008) and its follow-ups, particularly in motivating the use of “K” values for change detection even in high-profile new experiments? The ongoing influence of Rouder et al. (2008) on theorizing and measurement could reflect the erroneous view that all-or-none models are proxies for hybrid or “mixture” models of visual working memory (Zhang & Luck, 2008). Importantly, as reviewed, this view is incorrect because mixture models postulate variations in memory precision and differ conceptually and formally from all-or-none models of memory (for extended discussion of this point, see Robinson et al., 2020; Williams et al., 2022). In fact, comparing mixture and continuous resource models can be extremely difficult because, unlike all-or-none models that make qualitatively different predictions than resource models, mixture models can also predict curvilinear ROCs. Therefore, within the broader empirical and theoretical work on visual working memory, the high-impact study of Rouder et al. (2008) is an exception because it is one of few formal modeling studies that provides support for strictly all-or-none models of visual working memory, and, thus, almost single-handedly supports an extremely widespread application of all-or-none measures (“K” values) and views on the architecture of working-memory (e.g., Cowan, 2001).

### Critical assessment and replications by Donkin et al. (2014)

To address why Rouder et al. (2008) found evidence for the all-or-none model – despite its lack of support in other tasks — Donkin et al. (2014) conducted direct and indirect replications of Rouder et al. (2008). Importantly, the replications and analyses of Donkin et al. (2014) were designed to address several of the major methodological and analytic limitations discussed in this article, most notably the use of non-diagnostic data and biased model recovery metrics. In their critical follow-up experiments, Donkin et al. (2014): (1) reran the Rouder et al. (2008) experiment with a nearly identical design and larger sample size (> 90 participants instead of 23; Experiment 1); (2) ran a high-powered replication at the level of trials (> 2,000) and increased the number of base-rate conditions (from 3 to 5) to increase the chance that empirical ROC functions were diagnostic for testing the models (Experiment 2); and (3) ran an experiment with a larger number of base-rate conditions but a single set size to check the robustness of the modeling results (Experiment 3). Critically, the authors also assessed the diagnosticity of model comparison metrics using model recovery. Donkin et al. (2014) reported evidence for all-or-none models in their first two experiments in which they varied memory load (Experiments 1 and 2), as in the study of Rouder et al. (2008), but not in one experiment in which memory load was held constant throughout the experimental session (Experiment 3), and the authors noted that evidence for either model within and across experiments was ambiguous (“*Taken together, the results of the four experiments provide a rather mixed message regarding whether one should prefer the DS or the SDT model.*” p. 2110). These authors considered several explanations for their results, including non-diagnostic data and restricted model assessment, and that people strategically change how they maintain visual memory representations based on their expectations about memory load (Donkin et al., 2016), encoding whole instead of partial item information when memory load is unpredictable.

Some alterantive proposals are also that when visual memoranda are stored in memory, there is always some noise associated with the representations; however, when the changes are salient or “big” (e.g., the change happens across rather than within categorically distinct colors) as in Rouder et al. (2008), the amount of perceptual noise is insufficient to induce a confusion between the original item and the (changed) comparison probe, and this, consequently, reduces the contribution of noise on performance (Donkin et al., 2013; Nosofsky & Gold, 2016). Although these views provide a sophisticated framework for bridging inconsistencies across experimental paradigms, they have not received consistent empirical support in other change detection experiments that use salient changes (Robinson et al., 2020; Williams et al., 2022; Winiger et al., 2022), nor other forced choice paradigms that use mixed set size manipulations (e.g., Schurgin, et al., 2020; Wilken & Ma, 2004). Through this lens, results of Rouder et al. (2008) and Donkin et al. (2014) stand as an exception in the visual working memory literature. As discussed, this point is critical because these studies provide the dominant empirical support for all-or-none models and continue to motivate prominent measures of visual working memory limits (“K” values as a measure of capacity) in the field.

In general, such empirical inconsistencies can signal true, theoretically meaningful processing differences across paradigms, which may warrant revising core theoretical assumptions as part of routine theory development (Margolis, 1987). In the current context, results of Rouder et al. (2008) and Donkin et al. (2014) may require postulating that the effects of noise on memory representations differ as a function of experimental conditions (as proposed by Donkin et al., 2013; Nosofsky & Gold, 2016). Alternatively, inconsistencies across studies could also indicate that researchers failed to identify and test auxiliary assumptions. In this case, an empirical anomaly could be an artifact of limited methodology or analytic approaches, such as the methods used to construct ROCs and compare models, respectively, and, as such, not warrant revising the core theory.

In the remainder of the article, we test between these alternatives by directly re-examining auxiliary assumptions of Rouder et al. (2008) and building on the critical reanalysis of Donkin et al. (2014). We begin by using these studies and mainstream ideas from the metascience literature to illustrate how assumptions are made at different stages of theory testing, which can be practical or conceptual, and at different levels of a theoretical framework, which can be specific to a theory or common to each competing theory.

### Auxiliary assumptions at different stages of theory assessment

The view that auxiliary assumptions play a fundamental role across multiple stages of theory assessment has been discussed for some time (Duhem, 1954). The central role of auxiliary assumptions in theory testing follows from the fact that researchers must bridge the “deductive gap” between a core theory—such as the view that visual memory representations are stored in an all-or-none fashion versus continuously— and empirical observations (Suppes, 1966; for an in-depth discussion of how auxiliary assumptions bridge the “deductive gap,” see Kellen, 2019).

**Fig. 2:**
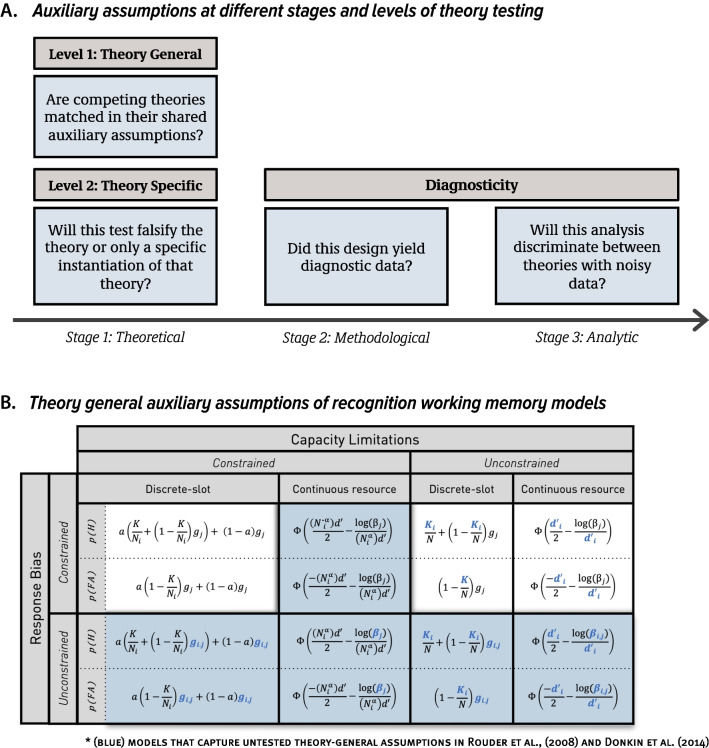
A schematic of assumptions made when testing psychological theories. (**A**) Theory general assumptions (top of Panel A) are those that can apply to any of the contending theories and encompass both core and auxiliary assumptions. Theory specific assumptions apply only to specific theories and encompass core and auxiliary assumptions. Auxiliary assumptions are also made at methodological and analytic stages of theory testing, and bear on the adequacy of methodology and analyses to discriminate between competing models. (**B**) The full factorial set of all-or-none and continuous resource models matched on each of the theory general auxiliary assumptions. Previous work considered only a subset of these (white cells), while not testing the remaining subset of possible models (blue cells), therefore, confounding auxiliary assumptions with core ones

First, auxiliary assumptions are made on a conceptual level because, on their own, basic theoretical propositions are underspecified. For instance, researchers must determine how to instantiate the view that visual working memory consists of “item limits” or “resources” as computational models that can be tested via ROC analysis. These computational models carry their own conceptual and parametric assumptions (see right panel of Fig. 2A). Some of these model-based auxiliary assumptions are *theory-specific*, meaning they apply uniquely to a particular theory. A popular example of what is commonly viewed as a theory-specific auxiliary assumption is that the distribution of memory strengths in continuous resource models is Gaussian in form (Wickens, 2001), which is instantiated with Gaussian signal detection models. This is often viewed as an auxiliary assumption (e.g., Kellen & Klauer, 2015; Rouder et al., 2010) because there are many types of continuous distributions (e.g., Gaussian, gamma, log-normal, etc.), and rejecting one of these does not rule out continuous resource theory as a whole.^1^

In addition to theory-specific auxiliary assumptions, there are theory-general auxiliary assumptions, which can apply to each of the contending theories. An example of a theory-general auxiliary assumption in the context of visual memory research is how processes, such as response bias and memory capacity, vary as a function of memory load in change detection tasks. For instance, one assumption made by Rouder et al. (2008) and Donkin et al. (2014) is that response bias parameters remain constant as a function of memory load, that is, the number of items that people must remember. Although this assumption can hold for both all-or-none and resource models, it does not follow logically from these models and is not core to the central question of whether ROC curves are linear or curvilinear.

Furthermore, as we discuss in greater detail below, signal detection models are compatible with other processing assumptions, including the view that people use strength of evidence rather than a likelihood ratio rule to make memory decisions. This strength of evidence decision rule would predict changes in response bias as a function of task difficulty, and therefore, memory load. This too suggests that fixing response bias parameters across memory load conditions may have not provided the best testbed for the signal detection model. To summarize, Rouder et al. (2008) and Donkin et al. (2014) examined a restricted subset of plausible all-or-none and continuous resource models and did not assess alternatives to each of these theory-general auxiliary assumptions. That is, the authors did not test the full set of auxiliary assumptions regarding how processes, such as response bias and capacity, vary as a function of memory load (see unexamined model variants in Fig. 2B). To emphasize, although none of these theory-general assumptions are core predictions of all-or-none and continuous resource theories, it is important to consider each of them to ensure that auxiliary theoretical assumptions are not conflated with core ones.

In addition to theoretical auxiliary assumptions, researchers also make practical auxiliary assumptions that bear on their choice of methodology (left panel of Fig. 2A). This step in theory assessment involves determining whether an experiment is likely to yield valid and reliable measures of central cognitive processes. In the current context, ROC analysis is the dominant modeling approach in the study of recognition memory (e.g., Wixted, 2007; Yonelinas & Parks, 2007) and involves comparing the relative fit of empirical ROCs, obtained with a specific methodology, to the theoretical ROCs predicted by each model. Rouder et al. (2008) and Donkin et al. (2014) used a base-rate manipulation to construct empirical ROCs, and, therefore, rely on the methodological assumption that this manipulation will yield ROC data that fall within a diagnostic region of ROC space – one that would allow them to discriminate between the linear and curvilinear ROC functions predicted by all-or-none and resource models, respectively (Fig. 1C). This assumption is auxiliary because it does not follow logically from either discrete or continuous resource theories. Importantly, previous work suggests that this assumption may, in fact, not hold (Dube & Rotello, 2012). That is, as pointed out by Donkin et al. (2014), participants may be insufficiently sensitive to all levels of a base-rate manipulation and, consequently, empirical ROCs can be noisy and/or restricted in range such that they are equally compatible with both models (gray region of Fig. 1C), making it difficult or impossible to discriminate between the models in practice. As such, it is critical to verify the auxiliary assumption that the methodology used to link theory to empirical observations is sound.

Finally, researchers make practical auxiliary assumptions when they choose how to analyze data and what metrics to use to draw inference. In the computational modeling domain researchers’ inferences are based on model comparison, and each model comparison approach rests on its own auxiliary assumptions that require validation (Lee et al., 2019). Rouder et al. (2008) and Donkin et al. (2014) apply a mainstream approach that involves comparing models based on their fit to data, using Akaike Information Criteria (AIC) and Bayesian Information Criteria (BIC) model comparison metrics. These metrics capture the goodness of a model’s fit to data, while penalizing its flexibility based on its number of parameters. Importantly, as also pointed out by Donkin et al. (2014), these model comparison metrics have been criticized because model flexibility is jointly determined by a model’s number of parameters and its functional form, and, therefore, penalizing a model solely based on its number of parameters may not always be appropriate (Myung et al., 2009). (To address this problem, Donkin et al., (2014) used landscaping analyses, though, as pointed out by the authors, these also showed ambiguous support for both models and, as also pointed out by these authors, model comparisons were not implemented when models were matched on their ‘theory general’ auxiliary assumptions.) Accordingly, it is critical to verify that AIC and BIC can be used to draw inferences from the data and reliably recover each of the generative models given the models and characteristics of the Rouder et al. and Donkin et al. data and methodology (Lee et al., 2019). 

## Reanalysis

In the following sections, we apply these ideas to re-evaluate the results of Rouder et al. (2008) and Donkin et al. (2014). For ease of exposition, we divide our reanalysis into three “steps,” which can be adapted by researchers in their assessment of their own or others’ work. The first step involves testing methodological and analytic auxiliary assumptions. We approach this problem via model recovery analysis, a well-known simulation-based approach that provides insight into whether a set of models could be recovered in practice given characteristics of the data (Lee et al., 2019). This model recovery analysis provides insight into whether the methodology yields diagnostic data and whether the model comparison metrics are well calibrated to recover each of the models. To preview, we find that these practical assumptions were violated in the original studies.

In the second step of our reanalysis, we examine whether diagnostic hypothesis-testing conditions can be found by considering the full set of theory-general auxiliary assumptions, matching all-or-none and continuous models on these assumptions, and implementing model recovery analysis to compare how recoverable each model is given the data and model comparison metrics. The final step involves testing the central hypothesis and drawing inference under these diagnostic testing conditions. We highlight that, while the details of our reanalysis are specific to these visual recognition memory studies, the analytic tools we use, such as model recovery simulations as well as our conceptual reanalysis of theoretical auxiliary assumptions, generalize across research domains.

### Step 1: Reassess auxiliary assumptions from original studies

We begin our assessment of Rouder et al. (2008) and Donkin et al. (2014) by examining whether there are signs that key auxiliary assumptions are violated in the original analysis. To this end, we implemented model recovery analysis to determine whether the data in each experiment of Rouder et al. (2008) and Donkin et al. (2014) as well as model comparison metrics could allow us to correctly recover the two best performing all-or-none and continuous resource models if they were the true generative models. We also reanalyzed the original data by assessing how models fit to data at the level of individuals, instead of the aggregate, to check the robustness of the modeling results.

#### Description of models

The two best performing models in Rouder et al. (2008) – and the two models assessed by Donkin et al. (2014) – were the all-or-none model with the attention lapse parameter, in which capacity, attention lapse, and response bias parameters were fixed across set sizes, and the equal variance likelihood ratio rule signal detection model, in which the resource parameter (*d’*) was free to vary across set sizes and response bias parameters were fixed across set sizes. The predicted hits and false alarms (FA) for the attention lapse, all-or-none model are shown in Eqs. 1a and 1b:

1a $$p(Hit) =a(K/{N}_{i}+(1-K/{N}_{i}){g}_{j})+(1-a){g}_{j}$$

1b $$p(FA) =a(1-K/{N}_{i}){g}_{j}+(1-a){g}_{j}$$

where the probability of a hit is the joint probability that people are paying attention to the display with probability $a$ and the probed item is in memory with probability $K/{N}_{i}$ – where $K$ denotes memory capacity and ${N}_{i}$ denotes the total number of items in condition $i$ and if an item is not in memory $(1-K/{N}_{i})$ and people correctly guess with probability ${g}_{j}$ in base-rate condition $j$ that the probed item changed. A hit can also occur if people are not paying attention to the display on a given trial $(1-a)$ and correctly guess that the probed item changed. The probability of a false alarm is the probability that people are paying attention ($a$) to the display but the item is not in memory and they incorrectly guess that the probed item changed $(1-K/{N}_{i}){g}_{j}$ or the probability that they are not paying attention to the display and incorrectly guess that the probed item changed $(1-a){g}_{j}$.

The equal variance likelihood ratio rule signal detection model postulates that distribution of memory strengths generated on signal (change) trials is normally distributed with unit variance and mean $d^{\prime}$, and the distribution of memory strengths generated on noise (no change) trials is normally distributed with unit variance and mean zero. Furthermore, the model postulates that people infer the probability of a change based on the likelihood ratio between these two distributions, given an observed memory strength signal $x$. The decision rule for this model as well as the derivations for the probability of hits and false alarms is shown in Eqs. 2a, 2b and 2c, respectively:

2a $$\phi (x-{d}_{i}^{\prime})/\phi (x)>{\beta }_{j}$$

2b $$p(Hit) = \Phi ({d}_{i}^{\prime}/2-log({\beta }_{j})/{d}_{i}^{\prime})$$

2c $$p(FA) = \Phi (-{d}_{i}^{\prime}/2-log({\beta }_{j})/{d}_{i}^{\prime})$$

where $\phi$ and $\Phi$ is the probability density and cumulative density of the normal distribution, respectively, ${d}_{i}^{\prime}$ is the mean of memory strength signals in set size condition $i$, and ${\beta }_{j}$ is the decision criterion for responding change in base-rate condition $j$.

#### Details of analysis

**Fig. 3:**
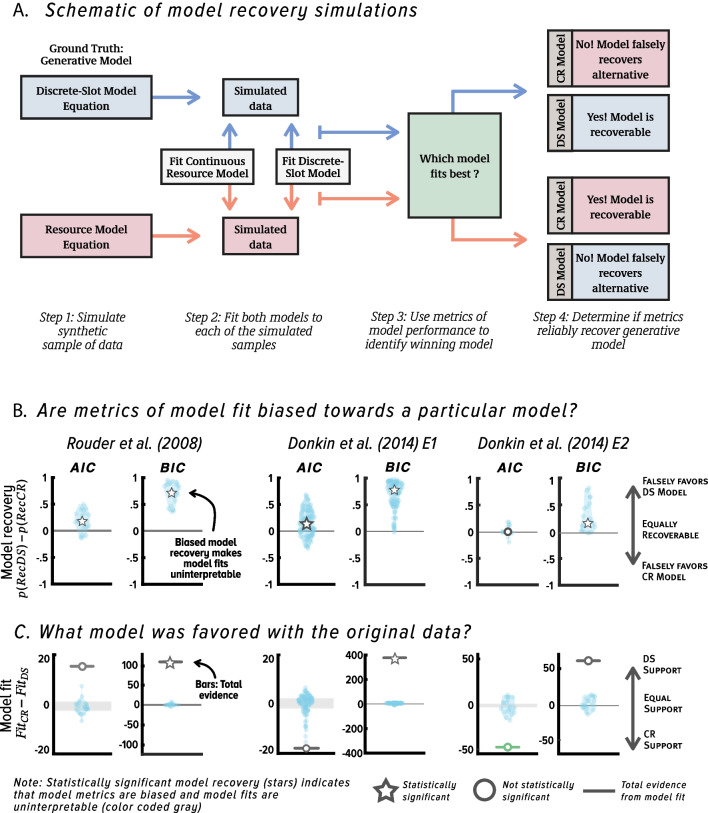
(**A**) Schematic of model recovery analyses, which can be used to assess diagnosticity of model recovery metrics and data in formal model comparison. (**B**) Results of model recovery reanalysis of Rouder et al. (2008) and Donkin et al. (2014) experiments with the original restricted set of all-or-none and continuous resource models. Model recovery results are quantified as the difference between $p(Rec DS)$ and $p(Rec CR)$; scores close to zero indicate that each model was recovered comparably, and scores closer to 1 (-1) indicate the continuous resource (all-or-none) model was not recovered as reliably as the all-or-none (continuous resource) model (see *Results* for further technical details). Circles and stars denote medians. (**C**) Results of model fits to original data. Note that when model recovery results are significant (denoted with white star in Panel B), this means that metrics of model fit are biased, or non-diagnostic and cannot be used to discriminate between the competing models. To emphasize this, model fit symbols (circle, star, and evidence bar) are coded in gray and green when results of model fit are uninterpretable and interpretable, respectively. Circles and stars denote total summed evidence

We used model recovery analysis to assess whether either of the two model comparison metrics (AIC or BIC) could correctly recover each of these two models if they had in fact generated the data, given characteristics of data from Rouder et al. (2008) and Donkin et al. (2014). Model recovery is recognized as a fundamental part of best practices in computational modeling because it provides an independent way of verifying whether each of the competing models would be correctly identified as a winning model under the hypothetical scenario that it is the generative model (Heathcote, et al., 2015; Lee et al., 2019; Wagenmakers et al., 2004; Zilker, 2022). As shown in Fig. 3A, model recovery analysis involves simulating data directly from the model equations, fitting the generative and each competing model to the simulated data, and evaluating which model is the best performing model with each metric of model fit (Heathcote, et al., 2015; Lee et al., 2019; Wagenmakers et al., 2004). Diagnostic metrics of model fit will correctly recover the true data-generating model, whereas non-diagnostic metrics will incorrectly favor a model that did not actually generate the data. In the context of our reanalysis, model recovery analysis provides insight into whether BIC or AIC could reliably recover both all-or-none and continuous resource models had they generated the data.

When validating model performance metrics via model recovery simulations, it is important to do so while replicating key aspects of the study design. For instance, models might be recoverable in the limit but not with noisy samples of data; therefore, simulated samples of data should have the same number of observations as the original samples. Likewise, the relative fit of the model will vary depending on the estimated parameter values; therefore, when implementing model recovery, it is important to ensure that generative models are recoverable given the best fitting parameter estimates within a given data set. As highlighted by Donkin et al. (2014), this approach helps address the question of whether metrics of model fit are diagnostic given the specific methodological and analytic approach chosen by the researcher. 

All modeling and analyses used data from Rouder et al. (2008) and Donkin et al. (2014), and were implemented in Matlab (for code see the Open Science Framework at: https://osf.io/mg63r/). Models were fit to data using Maximum Likelihood Estimation, by minimizing the negative log likelihood using the fmincon minimization algorithm. For the model recovery analysis, we simulated data 100 times from each model using parameter estimates from each of the participants in the three studies (a total of 27,600 simulations across participants and studies). We quantified model recovery reliability for each model by calculating the probability of recovering the correct generative model based on AIC or BIC across these simulations. For example, $p(Rec DS)=1$ and $p(Rec CR)=1$ means that the probability of recovering the all-or-none discrete-slot and continuous resource models, respectively, with a given metric is highly reliable for a participant within a given experiment. Relatively lower scores for one of the models indicate that the model was not recovered as reliably. 

To ease interpretability, we show the results of model fits and model recovery analyses graphically in Fig. 3B. For completion, we also list all values of model fit and best fitting parameter estimates that were used for model recovery in Table 1 and show the values of the model recovery metrics for each model comparison in Table 2. We found that the continuous resource model was essentially unrecoverable across the three studies when BIC was used to compare models. The version of the resource model tested by these authors has more parameters than the all-or-none model, so these results with BIC align with prior work in which BIC was incorrectly biased towards models with fewer parameters (e.g., Robinson et al., 2021; van den Berg et al., 2014). We also found that AIC failed to reliably recover the continuous resource model in the Rouder et al. (2008) study and in Experiment 1 of Donkin et al. (2014). The only exception was in Experiment 2 of Donkin et al. (2014), in which both models were recovered with equal reliability.

**Table 1:**
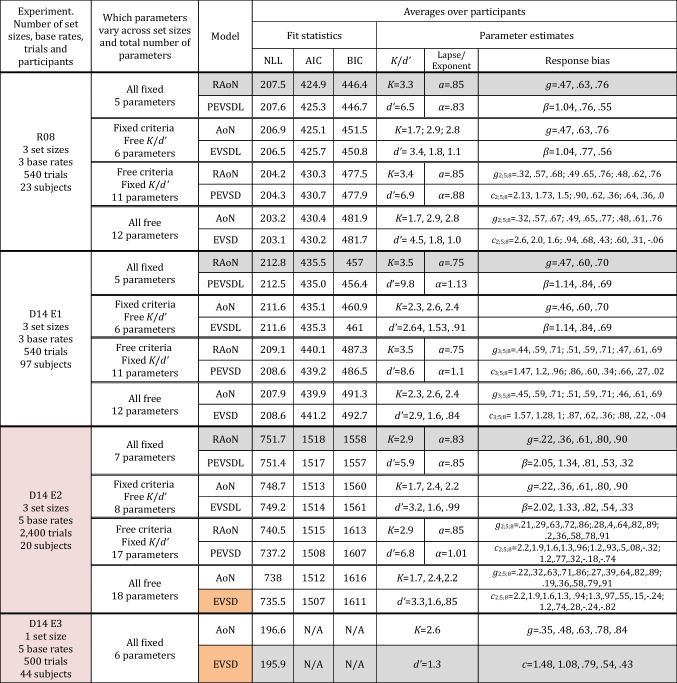
Models fit and best-fitting parameters. Summary of results of model fits from the Rouder et al. (2008) study (R08) and Experiments 1 (E1), 2 (E2), and 3 (E3) from Donkin et al. (2014) (D14). The summary includes results from a comprehensive set of model variants where all parameters were fixed across memory load conditions, criteria were free to vary and capacity/resource parameters were fixed and vice versa, and all parameters were free to vary across memory load conditions

RAoN refers to the Rouder version of the all-or-none visual working-memory (VWM) model (with an attention parameter), AoN refers to the standard all-or-none VWM model. PEVSD and PEVSDL refers to the power law version of the signal detection model where *d’ *is constrained to vary across memory load conditions via a power law, with the strength of evidence and likelihood ratio rule, respectively, EVSDL refers to the likelihood ratio rule signal detection model where *d’* is free to vary across memory load conditions and response criteria are fixed, and EVSD refers to the classic signal detection model where all parameters are free to vary across experimental conditions. Rows shaded in gray denote the models that were reported as best performing models in the original studies, and cells shaded in orange denote models that were recoverable and best performing in the current reanalysis

**Table 2:**
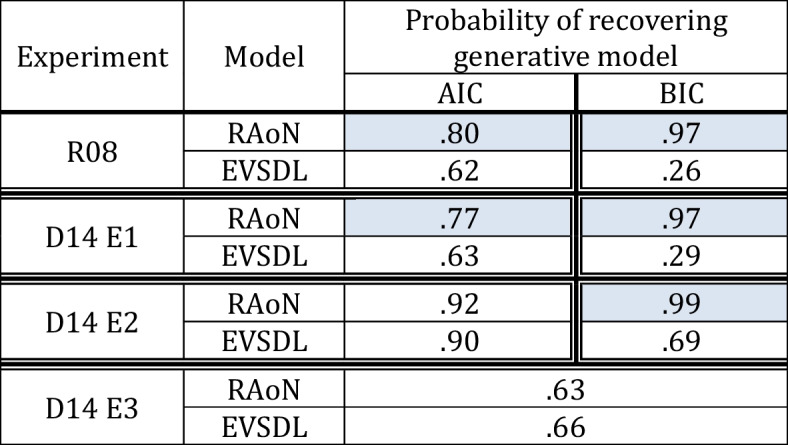
Results from model recovery for AIC and BIC with the restricted set of models used by Rouder et al. (R08) and Donkin et al. (E3)

Proportions denote the average number of times a given model was recovered out of 100 simulations per each model and participant. Values closer to 1 indicate that a given model was recovered perfectly across 100 simulations, and values closer to zero indicate that a given metric was biased towards the alternative model. Cells shaded in blue denote instances where metrics of model fit were significantly biased towards a given model based on paired t-test comparisons across participants (*p*s < .001). To summarize, in this set of model recovery analyses, AIC and BIC were significantly biased towards the Rouder et al. all-or-none model in the original Rouder et al. study as well as Donkin et al.’s Experiment 1, and BIC was biased towards the Rouder et al. all-or-none model in Donkin et al.’s Experiment 2. AIC and NLL were not biased towards either model in Donkin et al.’s Experiment 2 and Experiment 3, respectively; however, in these experiments metrics of model fit provided either ambiguous support or favored the resource model (see main text for details on model comparisons)

Next, we evaluated results from fitting this set of models to the real data at the level of individual participants. Our reanalysis of the original data revealed, surprisingly, that neither model received strong support (Fig. 3C) at the level of individual participants (a result also noted by Donkin et al., 2014). Although BIC favored the all-or-none model, which has fewer parameters, AIC did not statistically favor either model at the level of individual participants. Therefore, even though BIC favored the all-or-none model in the data, these results are inconclusive because model recovery shows that BIC tends to favor the all-or-none model even if the continuous resource model generated the data, that is, it could not recover this resource model in principle.

Together, we found that the original results are ambiguous as to which is the best performing model. This follows because we do not know how all-or-none and continuous resource models would compare if metrics of model fit could recover each of these models with equal reliability, and if we had considered the full scope of theory-general auxiliary assumptions when comparing models. Therefore, this reanalysis indicates that, in fact, basic methodological and analytic auxiliary assumptions in Rouder et al. (2008) and Donkin et al.’s (2014) Experiment 1 were violated. Model recovery shows that the data and/or model metrics in these studies were not well calibrated to compare all-or-none and continuous resource models and, furthermore, the original data did not reliably favor either model across participants. To summarize, the goal of our first reanalysis was to assess these studies for signs that key untested auxiliary assumptions were violated, and we found that they were.

### Step 2: Find diagnostic testing conditions

In our second reanalysis, we directly assess which auxiliary assumptions are violated, and look for conditions that provide a diagnostic testbed for these models. To this end, we considered the full factorial set of all-or-none and continuous resource model variants, instead of the restricted subset considered by Rouder et al. (2008) and Donkin et al. (2014). That is, we consider model recovery and fitting results when all-or-none and continuous resource models are matched on each of their theory-general auxiliary assumptions. In this context, each of the all-or-none and resource models has four variants, which reflects the full set of models crossed on their auxiliary assumptions regarding how response criteria and capacity or resources behave with changes in memory load (Fig. 2B).

This reanalysis has two advantages. First, it lets us test the auxiliary methodological assumption that these studies yielded diagnostic data (left panel of Fig. 2A). We do so by using model recovery and examining whether we can reliably identify a winning model under conditions where all-or-none and continuous are matched on their number of parameters. Second, it meets the conceptual criterion of comparing each model when they are matched on their theory-general auxiliary assumption and, therefore, their scope (right panel of Fig. 2A).

#### Description of models

In addition to the all-or-none model (Eq. 1a and 1b) and continuous resource model (Eq. 2a, 2b, and 2c) tested by Rouder et al. and Donkin et al., we include the following model variants. First, we test versions of the original models in which response bias parameters are free to vary across set sizes. This auxiliary assumption was untested by Rouder et al. (2008) and Donkin et al. (2014) (although Donkin et al. proposed this as a further follow-up), but warrants assessment for two reasons. The first reason is because the question of whether ROCs are linear (in line with all-or-none models) or curvilinear (in line with continuous resource models) is independent of whether response bias parameters are free to vary across memory load sizes or not. The second reason is that fixing response bias in the signal detection model only makes sense through the lens of a very specific assumption, which is that people use the likelihood ratio between signal and noise distributions rather than strength of evidence to make decisions in recognition memory tasks. This assumption is also ancillary in the context of comparing how people store information in visual working memory. Furthermore, a recent study provides evidence against the likelihood ratio signal detection rule (see Hu et al., 2023), indicating that evaluating alternative decision rules is tenable.

Importantly, prior work indicates that if people use a strength of evidence instead of likelihood ratio decision rule, they are more likely to set a conservative response criterion with increasing task difficulty (e.g., Benjamin & Bawa, 2004; Brown et al., 2007; Robinson et al., 2020). In the current context, this view predicts that people become more conservative in responding “no change” when memory load increases (a pattern we also found in these data). Together, there are principled reasons to consider variants of models in which criteria are allowed to vary freely across experimental conditions.

In addition, we assess two additional versions of the all-or-none model in which capacity is free to vary across set size, both with free and fixed response bias parameters across set sizes. The equation for this model is equivalent to the standard all-or-none model for single probe change detection tasks, and its predicted hits and false alarms are shown in Eqs. 3a and 3b, with notation identical to that used in Eqs. 1a and 1b:

3a $$p(Hit) = K_{i}/{N}_{i}+(1-K_{i}/{N}_{i}){g}_{j}$$

3b $$p(FA) =(1-K_{i}/{N}_{i}){g}_{j}$$

Finally, we also tested variants of a signal detection model where the resource parameter ($d^{\prime}$) was constrained to vary across set sizes, with both free and fixed response bias parameters across memory load conditions. These variants of the signal detection model were motivated by previous evidence that resources may change via a power law as a function of set size (e.g., Schurgin et al., 2020). Including this model allowed us to test a wider range of signal detection models as well as explore how signal detection models compare to all-or-none models under conditions where both theories predict that visual working memory limits are constrained to vary in a principled way across set sizes (via the power law and attention lapse parameter, respectively). The Equation of the power law signal detection model is identical to that shown in Eqs. 2a–2c, with the caveat that the $d^{\prime}$ is fixed across set sizes and is weighted by the number of items in a given memory load conditions ($N$), which is raised to a power $\alpha$, an additional parameter that is fixed across memory load conditions ($d^{\prime}{N}^{-\alpha })$.

#### Details of analysis

The analytic approach for implementing model recovery and assessment of model fit was the same as the one used in *Step 1*. The main critical difference is that our model recovery analysis and assessment of model performance was focused on pairs of models that were matched on their theory-general assumptions and number of parameters.

#### Results

**Fig. 4:**
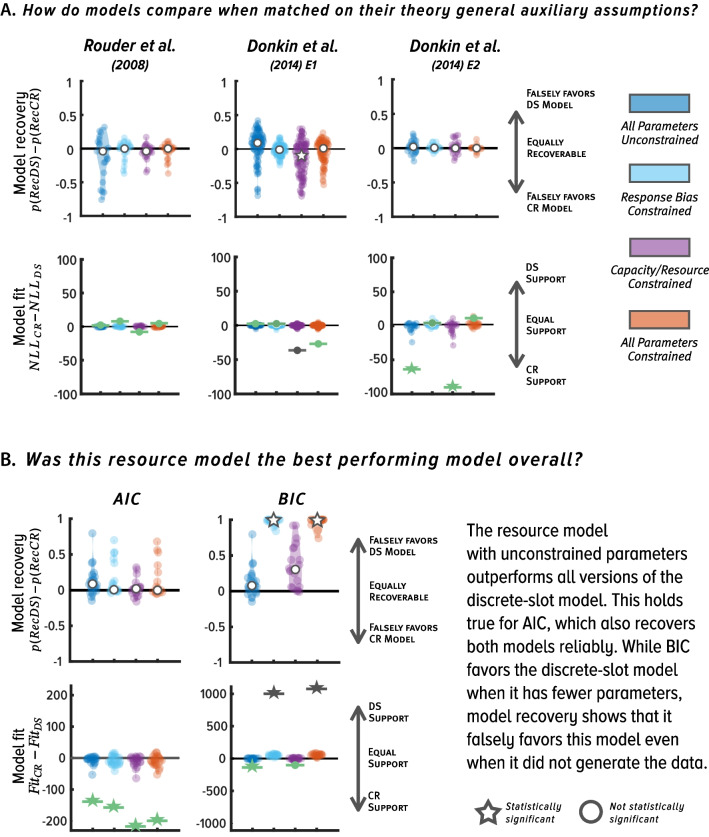
(**A**) Results of model fit and recovery with the full factorial set of all-or-none and continuous resource models when these are matched on their theory-general auxiliary assumptions. Comparisons are made only between “matched” pairs of all-or-none and continuous resource models; these have the same number of parameters and are compared with the negative log likelihood (NLL). When models are matched on theory-general auxiliary assumptions, they all fit the data equally well, except in Donkin et al. (2014; Exp 2), where the unconstrained resource model outperforms the unconstrained all-or-none model. (**B**) Top: Results of comparing the best fitting resource model to all variants of the classic discrete slot model in Experiments 2 of Donkin et al. (2014), where empirical receiver operating characteristics (ROCs) span a wider range of ROC space. We find evidence for the continuous resource model using AIC, which is the only well-calibrated (unbiased) model comparison metric, as shown by the model recovery simulations (bottom). Circles and stars in model recovery denote medians. Circle and stars in model fits denote total summed evidence and are green and gray, when model recovery shows that model comparison metrics are diagnostic and non-diagnostic, respectively

For simplicity, we report the negative log likelihood for all model comparisons because the matched models have the same number of parameters, and identical conclusions would be drawn with AIC and BIC. Table 3 summarizes results from model recovery analysis for each model, and Fig. 4A shows results of model fits and recovery graphically. Using model recovery, we found that each variant of the all-or-none and resource model was generally recovered with equal reliability when it was tested against its matched counterpart in all experiments. This means that we can use metrics of model fit to compare each of the model pairs and draw inferences about which is the best performing model. Critically, in these model comparisons we found that both in Rouder et al.’s (2008) study and Donkin et al.’s (2014) Experiment 1, all-or-none and continuous resource models fit the data equally well. That is, there was no statistically significant evidence for a best-performing model in these studies.

**Table 3:**
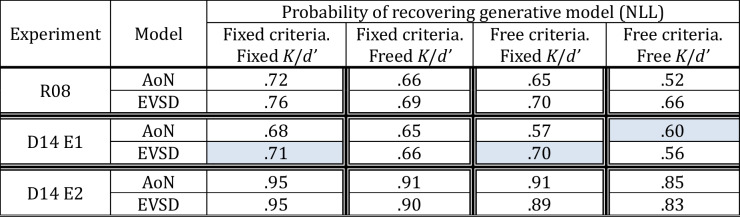
Results from model recovery using negative log likelihood (NLL), where all-or-none and resource models are matched on their theory-general auxiliary assumptions and number of parameters

Proportions denote the average number of times that a given model was correctly recovered out of 100 simulations per each model and participant. Values closer to 1 indicate that a given model was recovered perfectly across 100 simulations, and values closer to zero indicate that a given metric was biased towards the alternative model. Cells shaded in blue denote instances where metrics of model fit were significantly biased towards a given model based on paired t-test comparisons across participants (*p*s < .05). To summarize, NLL was biased towards the all-or-none model in Donkin et al.’s Experiment 1 when all parameters were free to vary across set sizes, and towards the resource model when resource capacity parameters were fixed across set sizes. Metrics of model fit showed comparable support for both models in the original Rouder et al. study and Donkin et al.’s Experiment 1, indicating that these data cannot be used to draw inferences about which is the best performing model. Model recovery analyses with Donkin et al.’s Experiment 2, however, showed no model bias and NLL showed support for the resource model when comparing models with real data

Together, these results indicate that data in the Rouder et al. study and Experiment 1 of Donkin et al. were not diagnostic for discriminating between all-or-none and continuous resource models. That is, each model can be recovered with equal reliability, but each model also fits the data as well as its competitor, meaning that these data could not be used to test between this set of matched all-or-none and resources models in principle.

These data may be non-diagnostic because the data fall within a restricted section of ROC space, in which the curvature predicted by the continuous resource model is nearly linear and overlaps with the linear ROCs of the all-or-none model (Fig. 1C). Notably, this region of overlap may be wider when only three points are used to construct ROCs with noisy empirical data and may be exacerbated if participants are not sufficiently sensitive to all levels of the base-rate manipulation (Dube & Rotello, 2012; Krantz, 1969). Ultimately, this reanalysis with the full factorial set of models reveals that both models fit the Rouder et al. (2008) and Donkin et al. (2014) Experiment 1 data comparably, indicating that the empirical ROCs were non-diagnostic.

Importantly, in Donkin et al.’s Experiment 2, we also found that all models were generally recovered equally well and robustly across participants, indicating that these metrics were not systematically biased to favor either model. Critically, in this experiment we find that no variant of the all-or-none model outperformed resources models, but two variants of the resource model – untested by Rouder et al. (2008) or Donkin et al. (2014) – outperformed their all-or-none counterparts (Fig. 4A).

Collectively, these analyses and results illuminate which untested methodological and theoretical auxiliary assumptions were violated. Data from two experiments, including the study of Rouder et al. (2008), were non-diagnostic for testing between all-or-none and continuous resource models. This follows because model recovery results indicate that metrics of model fit (LL) can recover each model with equal reliability, however, when fitting models to real data, there is no “winning” model. In contrast, data from Donkin et al.’s (2014) Experiment 2 reveal provisional support for two variants of resource models, at least when these are tested against all-or-none models that are matched on their theory-general auxiliary assumptions and have the same number of parameters. Together, at a minimum, the results of this reanalysis indicate that these studies provide no support for all-or-none models of visual working memory and provisional support for the continuous resource model.

### Step 3: Test central hypothesis and draw inference

So far, we found that in the original Rouder et al. (2008) and Experiment 1 of Donkin et al. (2014), all-or-none and continuous resource models provide comparable fits to the data, indicating that data in these experiments were not diagnostic and could not be used to tell these models apart. We also found that in Donkin et al.’s (2014) Experiment 2, variants of the continuous resource model outperformed all-or-none models when these models were matched on their theory-general assumptions. Having identified a potentially diagnostic sample of data, and considered the full scope of all models, we can now ask: *Is there a variant of the continuous resource model that is the best performing model overall?*

#### Description of models

In the following section we examine whether the continuous resource model is the best performing model overall when comparing it across all theory-general auxiliary assumptions. To this end, we collapse across the full set of theory-general auxiliary assumptions and compare the best performing variant of the continuous resource model— in which the resource and response bias parameters vary free across memory load conditions— to all variants of the all-or-none model. We underscore that, while the best performing resource model has more parameters than some of its all-or-none counterparts, as before, we directly assess if and which metric of model fit can recover the generative model given this difference in parameters. That is, before making inferences from model comparisons, we use model recovery analysis to assess ancillary assumptions about the diagnosticity of data and model comparison metrics.

#### Details of analysis

The general analytic approach for implementing model recovery and assessment of model fit was the same as the one used in *Steps 1* and* 2*. The critical difference is that this reanalysis focuses on Donkin et al.’s (2014) Experiment 2 data because results of model recovery and fit revealed that these were the only diagnostic data in experiments where memory load was manipulated.^2^ As discussed, we also focused our model recovery analysis and assessment of model fit on the best performing continuous resource model and all variants of the all-or-none model to examine whether the resource model was the best performing overall.

#### Results

Table 4 summarizes results from model recovery and Fig. 4B shows modeling results graphically. When using BIC we found that it favored the all-or-none model in two instances in which these models had fewer parameters than the resource model, but we also found that BIC was biased towards these all-or-none models based on model recovery. Therefore, BIC does not provide a non-biased measure of model comparison in this context. Importantly, however, using model recovery we found that AIC was not biased towards the best-performing resource model (top panel of Fig. 4B) and when fitting the models to real data using AIC, we found that the resource model outperformed each of the all-or-none models in Experiment 2 of Donkin et al. (2014). Together, our assessment using AIC indicates that the superior performance of the best performing resource model does not reflect poorly calibrated metrics of model fit, or non-diagnostic data, but that it provides a better account of the data than its all-or-none counterparts (bottom panel of Fig. 4B).

**Table 4:**
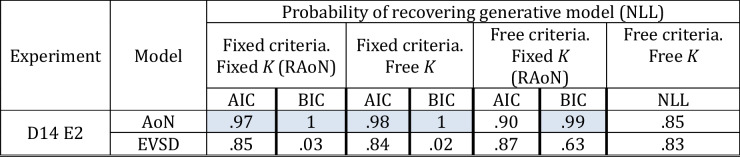
Results from model recovery for AIC and BIC when comparing the best performing signal detection model with parameters free to vary across memory load conditions to all variants of the all-or-none model

Proportions are the average number of times each model was recovered out of 100 simulations per each model and participant. Values closer to 1 indicate that a given model was recovered perfectly across 100 simulations, and values closer to zero indicate that a given metric was biased towards the alternative model. Cells shaded in blue denote instances where metrics of model fit were significantly biased towards a given model based on paired t-test comparisons across participants (*p*s < .05). To summarize, BIC was significantly biased towards the all-or-none model variants, however, AIC generally showed no bias. Model comparisons showed that the resource model outperformed all variants of the all-or-none model based on AIC. Together these results provide support for the continuous resource model

Collectively, our modeling results align with those of Donkin et al.’s (2014) Experiment 3, in which they also used five base-rate manipulations but held memory load constant across the experimental session. Our results further indicate that there is no need to postulate different strategies across visual working memory paradigms or conditions. The continuous resource model outperforms the all-or-none models when considering the full set of auxiliary assumptions and analyzing diagnostic data. It is likely that a critical aspect of Donkin et al.’s Experiment 3 is that the authors used a sufficient number of base-rate manipulations (five) to obtain diagnostic data, and— because the experiment only had a single memory load condition— the compared models were already matched on their number of parameters and theoretical scope. More broadly, these results show that once we identify diagnostic testing conditions, we find evidence for the completely opposite theoretical conclusion.

## General discussion

### Implications for theory and measurement in the working memory literature

We begin by discussing the implications of our results for research on visual working memory. Our results corroborate the view that continuous resource rather than classic all-or-none models best capture working memory processes in change detection. This finding is consistent with results obtained in visual continuous reproduction paradigms, which no longer consider the classic all-or-none model of memory as a plausible model of visual working memory (van den Berg et al., 2014; Zhang & Luck, 2008). Support for continuous resource models of visual working memory also corroborates results from a recent minimal assumptions critical test (Winiger et al., 2022), recent ROC analyses using confidence-based judgments (Robinson et al., 2020; Williams et al., 2022), and novel unifying framework of memory (Schurgin et al., 2020).

Our findings also indicate that the popular metric of performance ($K$) is not appropriate for measuring working memory processing in change detection tasks, because this model assumes linear ROCs (and no variation in “precision” or “strength”). More recent work by Williams et al. (2022) demonstrates that using this metric can lead to drastically different conclusions than metrics based on resource models and, therefore, the choice between them can have significant implications for researchers who use change detection tasks to quantify visual working memory limits. Collectively, our data fit with the more parsimonious view that there is no need to postulate different processing assumptions across experimental paradigms or experimental conditions. That is, classic all-or-none models are simply not supported in visual working memory at all. More broadly, our results underscore the point that measurement and theory mutually constrain each other (e.g., Kellen et al., 2021a), that the development of both proceeds in an iterative fashion (Chang, 2004), and that an important next step in the field is to focus on building robust theories of visual working memory while integrating new knowledge in a theory-driven manner (Brady et al., 2022; also see visual working memory “theory map” in Ngiam, 2024).

Finally, we note that throughout our reanalysis we used a restricted set of possible analytic approaches, particularly, maximum likelihood estimation to estimate best fitting parameters and AIC and BIC to compare models. We used these tools because, after matching models on their theory-general auxiliary assumptions, the modeling results were consistent across participants, indicating that there was no need to model individual differences (e.g., via hierarchical Bayesian modeling) (Lee et al., 2019). Furthermore, this analysis provides insight into whether we could replicate the results of Rouder et al. (2008) and Donkin et al. (2014) while keeping as true to the original analytic approaches as possible. Finally, we vetted these metrics with model recovery, which provides insight into whether model comparison metrics are diagnostic. That is, analytic tools that robustly recover the correct model in simulated data can be used to draw inference when they are fit to real data, and ones that do not correctly recover the data-generating model cannot. Through this lens, model recovery can be used to guide inference by providing a ground truth on whether a given analytic approach can recover the generative model in principle (e.g., Heathcote et al., 2015; Lee et al., 2019; Wagenmakers et al., 2004; Zilker, 2022).

### Theory assessment practices beyond visual working memory

In this section we connect our case study to other examples outside of the visual working memory domain to the broader literature on best practices in theory assessment. First, as previewed in the *Introduction*, Starns and colleagues (2019) illustrated the central role of auxiliary assumption in shaping inference by showing that researchers can reach fundamentally different conclusions even when analyzing the same set of data. These authors used a blind-inference procedure in which a group of recognition memory researchers were blinded to key independent variables in recognition memory studies and had to infer them using their preferred analytic techniques. Critically, these authors found that only slightly more than half of researchers reliably drew correct inferences from the same data. Similar findings were reported in the response-time modeling literature by Dutilh et al. (2019). Together, these articles indicate that researchers may vary significantly in the (tacit) auxiliary assumptions they make in basic stages of data analysis and inference, and these auxiliary decisions can drive qualitative differences in researchers’ conclusions.

Kellen et al. (2021b) provide one specific example of how researchers can introduce bias when comparing theories by considering a restricted set of theory-specific auxiliary assumptions. For instance, in the long-term memory domain researchers may test only a single parametric variant (e.g., Gaussian) of many possible signal detection models and generalize inferences from this test to signal detection theory as a whole. This practice can bias theory development and assessment because it ignores the full scope of the core theory and because researchers may have different priors on which auxiliary assumptions are tenable.

An important caveat to the Kellen et al. (2021b) example is recent evidence that the Gaussian parameterization of signal detection models can have a principled theoretical basis. Robinson et al. (2023) (see also Thompson & Singh, 1967) point out that, by Central Limit Theorem, the Gaussian distribution implies that people pool sensory evidence via averaging or summation to construct memory representations. These authors tested this prediction in the visual working memory domain and found converging evidence for the Gaussian (as opposed to Gumbel) signal detection model. Together, these articles highlight that what counts as auxiliary in one context may not carry over to another. One obvious reason for this is that debates spawn theoretical questions that can form novel, complimentary lines of inquiry. For instance, the question of how to construe the architecture of visual working memory can generate new questions regarding how – through the lens of resource theory – memory representations are “built-up” from sensory evidence. Such questions may only be testable through specific parameterizations of computational models.

The view that what counts as a core versus auxiliary may not be a static property of a theory raises the question whether it makes sense to distinguish between core and auxiliary assumptions at all. This issue was discussed in the decision-making domain by Zilker (2022), who used model recovery simulations to demonstrate that specific choice rules, which connect latent preferences to observed choices, can impact researchers’ ability to diagnostically compare mainstream decision theories, such as Expected Utility and Cumulative Prospect Theory as examples. More precisely, Zilker found that Expected Utility and Cumulative Prospect theory could not be identified when deterministic (and trembling hand), rather than variants of probabilistic (logit and probit) choice rules are used for decision problems that make quantitatively rather than qualitatively different predictions for the two theories. This result follows because deterministic trembling hand choice rules can capture qualitative differences between choices, but not graded differences between them. Based on these findings Zilker concludes: “...*assumptions that are conventionally considered auxiliary can shape predictions and inferences to a similar or even higher degree than assumptions that are conventionally thought to constitute the core of formal models. These insights cast doubt upon the conventional division between core assumptions and auxiliary assumptions in computational modeling and emphasize the potential pitfalls*.”

Zilker’s systematic reanalysis illustrates the important role of auxiliary assumptions in shaping inference, though we believe that, rather than providing a   challenge to the distinction between core and auxiliary assumptions, this analysis highlights the need for using diagnostic methodologies and stimuli (Broomell & Bhatia, 2014; Regenwetter & Robinson, 2017). The fact that some decision problems do not provide diagnostic testing grounds across a range of auxiliary assumptions calls for a need to use tests that can (Mayo, 2018), just like, by analogy, our finding that some base-rate manipulations may not yield diagnostic testing conditions for all-or-none and resource models requires identifying more sensitive testing conditions. Developing such critical tests hinges on distinguishing between what counts as core and ancillary to the theory.

More broadly, evidence that a theory is limited in scope because it outperforms competing theories under a restricted set of conditions may signal that the core theory requires revision. This point may also not challenge the distinction between core and auxiliary assumptions per se, but underscore the view that theory development is an inherently dynamic practice (Box, 1976), where new insights generate novel research questions that can reshape what counts as core and auxiliary. For instance, it may make sense for decision researchers to focus on which decision rules – deterministic or probabilistic – best characterize how people map latent preferences to responses, just like it may make sense for memory researchers to ask how people construct memory representations from sensory evidence. Provided researchers are transparent about their research goals (Simmons et al., 2011) through tools such as preregistration (e.g., Wagenmakers et al., 2012), this is a routine part of theory development.

Next, we consider recent work that promotes substituting or supplementing theory-driven approaches with “bottom-up” tools for scientific discovery. Dubova et al. (2023) used agent-based modeling to simulate the consequences of using theory-motivated versus random experimentation. In this work, artificial agents could either choose how to sample existing distributions of data based on theory-motivated reasons, such as the goal of confirming or falsifying a theory-based hypothesis, or sampled data at random or in exploratory fashion. Critically, the authors found that random and exploratory sampling yielded a better characterization of the data-generating distribution overall. This suggests that theory driven as opposed to random experimentation can lead to biased data sampling that distorts subsequent theory development and, moreover, that researchers may remain unaware of these consequences. In the current context, this work raises the question of whether, instead of increasing the rigor of theory assessment, a better move is to simply replace theory-driven approaches with bottom-up, data-driven approaches.

We believe that while bottom-up theory assessment tools can help significantly improve theory assessment, they cannot replace it. First, as pointed out by Dubova et al. (2023), these simulations do not provide a clear proxy for scientific experimentation because agents do not need to design the experiments themselves. Critically, neither experimental design nor measurement in psychology can proceed without theory. This is because in order to determine how to measure or manipulate processes such as “memory,” researchers must use theory to postulate the hypothetical construct itself (Kellen et al., 2021a, 2021b; van Fraassen, 2008), make assumptions about which and how a given independent variable might affect it, and which metrics provide the best approximation of it (Brady et al., 2022; Rotello et al., 2015). As such, theory is built into experimentation and measurement in psychology. Arguably, this point applies to most scientific disciplines: researchers will rely on theory as long as there is a need to provide an overarching explanation or description for phenomena (Devezer, 2023; Newell, 2012).

Furthermore, there are important examples of how theory-driven approaches have generalized beyond the laboratory. For instance, the application of signal detection theory in psychology has helped improve measurement practices in real-world eyewitness memory tasks (e.g., Wixted et al., 2018). At this stage, it is unclear how purely data-driven, atheoretical approaches could yield similar insights. Finally, as noted by Dubova et al. (2023), there may be critical, unexamined tradeoffs between bias and resource efficiency when using theory-based versus random approaches to experimentation.

Despite these potential limitations, we strongly agree that automatization (e.g., Yarkoni et al., 2021), randomized methods in experimentation and analysis (Baribault et al., 2018; Davis-Stober et al., 2024), and data-driven and simulation-based approaches (e.g., Peterson et al., 2021; Cavagnaro et al., 2010) might provide essential, supplementary tools for countering researcher’s biases in theory assessment. Our core message is that scientists should go beyond polarizing theory-based versus bottom-up approaches and focus on how to optimally integrate them (for related discussions, see Devezer, 2023; van Rooij et al., 2023). In addition to these tools, along with others, we believe that formal modeling can improve the rigor of psychological theories because its application forces researchers to be explicit and precise about the assumptions of their model and its basis theory (e.g., Grahek et al., 2021; Guest & Martin, 2021; Navarro, 2021; Oberauer & Lewandowsky 2019). Relatedly, assessing models based on their ability to generalize to new domains, rather than solely fit a sample of data, may help improve theory assessment by curbing post hoc theorizing (Busemeyer & Wang, 2000; Popov, 2023; Newell, 2012; Robinson & Steyvers, 2023; for recent applications of this approach in the visual working memory domain, see Robinson & Brady, 2023; Schurgin et al., 2020).

Importantly, as our case study illustrates, computational modeling studies should still be supplemented with a careful conceptual analysis that disentangles core and auxiliary assumptions. On the methodological and analytic side, like others, we promote that model recovery should be a standard practice in model comparison (Heathcote, et al., 2015; Lee et al., 2019; Wagenmakers et al., 2004; Zilker, 2022), even with models that are vetted in prior work.

## Conclusion

To summarize, a failure to identify and separate auxiliary from core theoretical assumptions can lead to the spurious rejection of a model and theory (Kellen et al., 2021a). As we show in the context of recognition theories of working memory, this can have profound and long-lasting effects on an entire research domain. Along with others, we believe that a major step towards improving theory testing is for social scientists to become more aware of the auxiliary assumptions they make at different phases of scientific inquiry, including measurement (e.g., Brady et al., 2022; Kellen et al., 2021b; Guest & Martin, 2021; Regenwetter et al., 2022a; Rotello et al., 2015; Williams et al., 2022), analysis (e.g., Dutilh et al., 2019; Starns et al., 2019), and conceptual theory assessment (e.g., Kellen et al., 2021b; Regenwetter et al., 2022b; van Rooij & Baggio, 2021). In this context, our article integrates these ideas and provides an illustrative guide for how researchers can identify and test auxiliary assumptions at different levels of theory assessment and stage of study design. We believe that increased focus on this practice – particularly if supplemented by feedback from action editors and reviewers – is a major step towards increasing the rigor of theory testing in psychology.

## Data Availability

All data are reanalyzed from a prior work and are available online at the following link: https://osf.io/mg63r/.

## Footnotes

1 We note that a Gaussian distribution can have a principled theoretical basis through the lens of Central Limit Theorem (Green & Swets, 1966), which is consistent with recent work showing that people pool sensory evidence to construct Gaussian visual memory-strength representations (Robinson et al., 2023). We revisit this point in the *General discussion*, where we discuss how, under some conditions, theoretical auxiliary assumptions can become core assumptions as part of theory development.

2 Donkin et al.’s Experiment 3 does not manipulate memory load and, as reported by the authors, this experiment also provides support for the continuous resource model.

## Code availability

Code is available at the following link: https://osf.io/mg63r/.
